# Sequence determinant and functional relevance of 8-oxoguanine RNA modification unveiled from foundation-model-based predictor

**DOI:** 10.1016/j.omtn.2026.102951

**Published:** 2026-05-14

**Authors:** Rong Xia, Jiahao Zhang, Xuan Wang, Jiongming Ma, Jiayi Li, Jionglong Su, Prudence Wong, Daiyun Huang, Jia Meng, Bowen Song

**Affiliations:** 1 Department of Public Health, School of Medicine, Nanjing University of Chinese Medicine, Nanjing 210023, China; 2 Department of Biosciences and Bioinformatics, Center for Intelligent RNA Therapeutics, Suzhou Key Laboratory of Cancer Biology and Chronic Disease, School of Science, Xi’an Jiaotong-Liverpool University, Suzhou 215123, China; 3 School of AI and Advanced Computing, XJTLU Entrepreneur College (Taicang), Xi’an Jiaotong-Liverpool University, Suzhou 215123, Jiangsu, China; 4 Department of Computer Science, Faculty of Science and Engineering, University of Liverpool, Liverpool L69 3BX, UK; 5 Institute of Systems, Molecular and Integrative Biology, University of Liverpool, Liverpool L7 8TX, UK; 6 Academy of Pharmacy, Xian Jiaotong-Liverpool University, Suzhou 215123, China

**Keywords:** MT: Bioinformatics, RNA modification, first 8-oxoguanine (o^8^G) predictor, deep learning, interpretable foundation model, motif discovery, genomic patterns, web server predictor

## Abstract

8-Oxoguanine (o^8^G) is an oxidative RNA modification that plays critical roles in cellular processes including stress responses and RNA integrity regulation. Notably, emerging evidence has implicated o^8^G modifications in cancer progression and development. However, research progress is limited by the lack of efficient high-throughput detection methods and computational tools for analyzing sequence distribution and regulatory mechanisms. Here, we present OBOE (foundation-model-based prediction of 8-oxoguanine sites), the first computational framework for o^8^G site prediction, developed by fine-tuning multiple pre-trained language models (DNABERT, RNABERT, BERT, and BioBERT). Benchmarking experiments demonstrated that OBOE significantly outperforms conventional machine learning methods, validating its superior capability in capturing RNA sequence features and modification patterns. Furthermore, we applied TF-MoDISCo to extract biologically meaningful motifs from model attributions, followed by validation of sequence similarity and enrichment using STREME and TOMTOM analysis. We identified recurrent GC-rich sequence motifs and CTC-like patterns associated with o^8^G modifications, suggesting potential *cis*-regulatory elements involved in oxidative stress responses. To facilitate model accessibility, we implemented two community resources: (1) a user-friendly web platform for online o^8^G prediction and (2) freely available source code and processed datasets.

## Graphical abstract

**Figure:**
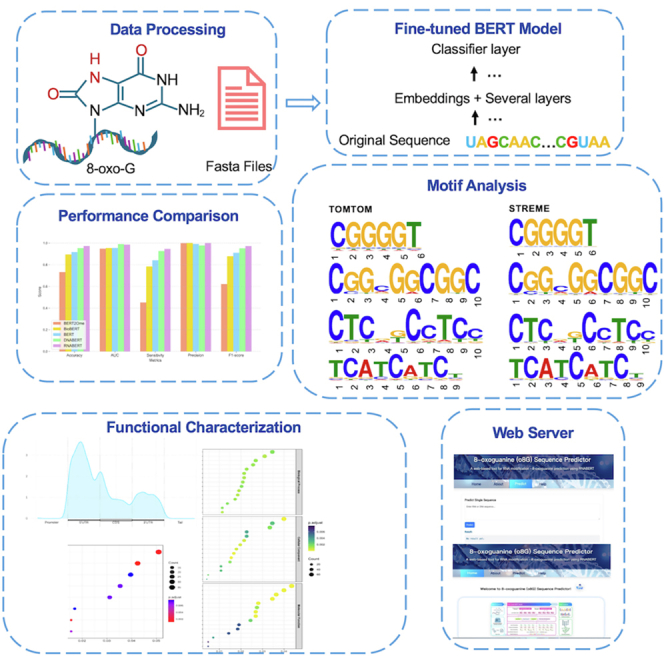

## Teaser

Xia and colleagues introduce OBOE, a foundation-model-based predictor for RNA 8-oxoguanine (o8G) modification sites. The framework outperforms conventional methods and identifies GC-rich sequence motifs linked to oxidative RNA modification, while an accessible web server enables genome-scale o8G site prediction.

## Introduction

The term “epitranscriptome,” referring to chemical modifications of RNA, represents a crucial layer of post-transcriptional regulation that influences diverse biological processes, including mRNA stability, translation efficiency, and stress response.1^1^^,^^2^ To date, over 170 types of chemical markers have been identified. Among these, 8-oxoguanine (o^8^G), an oxidative modification induced by reactive oxygen species (ROS), remains relatively understudied. Emerging evidence underscores the functional importance of o^8^G in preserving RNA integrity and modulating cellular responses to oxidative stress.3^3^^,^^4^ 8-oxoG in RNA not only compromises transcriptional fidelity and activates RNA decay pathways but also contributes to post-transcriptional regulation by remodeling RNA-RNA interactions, thereby functioning as a potential epitranscriptomic marker under oxidative conditions.5^5^ Critically, o^8^G modifications have been implicated in regulating cancer cell progression and development, representing a novel therapeutic target across multiple cancer types.6^6^^,^^7^ For instance, o^8^G-modified circular RNAs (circRNAs) suppress lung cancer progression through chaperone-mediated autophagy.8^8^ Additionally, o^8^G modifications can reprogram tumoral microRNA networks by altering their target specificity, thereby influencing tumorigenic pathways.9^9^^,^^10^ Collectively, these recent advances highlight the pivotal roles of o^8^G in cellular physiology and oncogenesis.^11^ However, in contrast to extensively characterized modifications such as N6-methyladenosine (m6A) methylation, research on o^8^G has been limited by the lack of efficient high-throughput detection methodologies and computational models for further deciphering its sequence distribution and regulatory mechanisms.

Recent advances in chemical labeling techniques have enabled transcriptome-wide profiling of o^8^G RNA modifications at exon-level resolution. In particular, ChLoRox-seq, a newly optimized antibody-free method that covalently labels o^8^G with biotin, allows specific enrichment and sequencing of oxidized regions with high specificity and reduced cost.1^12^ However, experimental approaches are often costly, labor-intensive, and time-consuming, thereby limiting comprehensive exploration of the biological functions of o^8^G modifications. Consequently, there is an urgent need to develop precise and efficient computational methods for predicting and analyzing o^8^G modifications.

To date, several computational frameworks have been successfully applied to epitranscriptome studies, achieving significant progress in prediction accuracy,^13^^,^^14^ interpretability,^15^^,^^16^^,^^17^ and biological relevance,^18^^,^^19^^,^^20^ demonstrating a strong foundation for the computational study of RNA modifications. One of the earliest tools for mammalian RNA methylation site prediction is SRAMP,^21^ which utilized sequence-derived features in a support vector machine (SVM) framework.^22^ Subsequently, WHISTLE improved prediction performance by incorporating genome-derived features into a machine-learning-based approach.^23^^,^^24^^,^^25^ With the advancement of deep learning,^26^ models such as Fan et al.’s hybrid framework^27^ and Luo et al.’s deep deposition model^28^ further enhanced performance by capturing complex sequence dependencies. Deep learning has also facilitated cross-species and cross-tissue modeling.2^29^^,^^30^ For instance, Xiong et al.3^31^ proposed a curriculum learning approach for multi-species prediction, while Wang et al.3^32^ extended modeling to single-cell resolution, enabling trajectory inference of methylation. Additionally, general platforms like OpenAc4C,^33^ MultiRM,3^34^ and RNA-ModX3^35^ were developed to predict multiple modification types simultaneously.^36^ Broad-spectrum tools such as ATTIC,3^37^ CircRM,3^38^ and comprehensive reviews^39^ further advanced the benchmarking and integration of diverse methodologies. Innovations like weakly supervised learning4^40^ and iterative feature engineering4^41^ also addressed challenges posed by low-resolution data. Despite these advancements, existing models typically rely on curated, labeled datasets for training, limiting their timely adaptation to emerging biological questions, such as the functional exploration of o^8^G modifications, which remain poorly characterized yet exhibit growing importance. Currently, no effective computational approaches exist to predict o^8^G sites or decipher their sequence determinants.

More recently, a growing number of foundation models in bioinformatics have emerged, leveraging extensive collections of protein, DNA, and RNA sequence data to extract evolutionarily conserved patterns that can be applied to various biological applications.^42^ A predominant architectural approach involves encoder-only Transformer networks trained through masked language modeling (MLM) objectives, including DNABERT4^43^ and RNABERT.4^44^ DNABERT employs its transformer architecture to derive comprehensive, generalizable knowledge from DNA sequences, enabling it to process downstream tasks such as promoter region prediction and identification of transcription factor (TF) binding sites. RNABERT demonstrates versatile capabilities in predicting RNA secondary structures, categorizing non-coding RNA groups, and characterizing novel transcripts, offering valuable structural insights into RNA biology. Several specialized models targeting RNA sequences have also been proposed. The Bert2Ome platform4^45^ combines BERT architecture with convolutional neural networks to achieve accurate detection of 2′-O-methylation sites, revealing important biological patterns. Similarly, BERT-m7G represents an innovative solution for high-accuracy prediction of m7G modifications in RNA sequences.4^46^

In this study, leveraging the powerful representation learning capabilities of foundation models and their success in various downstream biological applications, we proposed the first computational framework for o^8^G site prediction, OBOE (foundation model-based prediction of 8-oxoguanine site), by fine-tuning a diverse set of pre-trained language models, including DNABERT, RNABERT, BERT, and BioBERT. Benchmarking experiments demonstrated that OBOE achieves state-of-the-art performance in o^8^G identification, outperforming all baseline and tested models. Beyond accurate prediction, we conducted comprehensive analyses to interpret the learned sequence representations. Specifically, we applied TF-MoDISCo4^47^ to extract biologically meaningful motifs from model attributions, followed by validation of sequence similarity and enrichment using STREME4^48^ and TOMTOM4^49^ analysis. Our findings revealed recurrent and conserved GC-rich sequence patterns associated with o^8^G modification, suggesting potential *cis*-regulatory elements that may participate in oxidative stress responses.

**Figure 1:**
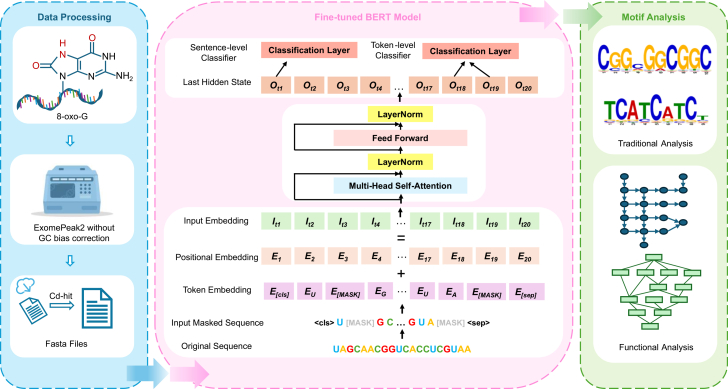
Overall workflow In our study, we analyzed raw sequencing data obtained through ChLoRox-seq from oxidatively stressed BEAS-2B human lung epithelial cells. For transcriptome-wide identification of o^8^G-enriched regions, we processed the data using the exomePeak2 pipeline with GC bias correction, as this approach is more appropriate for detecting non-consensus oxidative modifications such as o^8^G that exhibit diffuse distribution patterns. We developed OBOE, the first computational framework specifically designed for o^8^G site prediction, by fine-tuning pre-trained foundation models including DNABERT, RNABERT, BERT, and BioBERT to achieve optimal performance. To gain biological insights into o^8^G modification patterns, we implemented an interpretability pipeline combining TF-MoDISco for attribution analysis with STREME and TOMTOM for motif discovery and validation. Additionally, MetaTX was applied to obtain the transcriptome distribution o^8^G-containing peaks across transcripts. Functional enrichment analyses including Gene Ontology (GO) enrichment and Kyoto Encyclopedia of Genes and Genomes (KEGG) analysis were applied to further elucidate the biological functions associated with o^8^G.

Additionally, MetaTX analysis revealed that o^8^G modification is enriched in the 5′ untranslated region (5′UTR). An overview of the experimental and computational workflow is illustrated in Figure 1. To maximize model accessibility, we implemented two community resources: (1) a lightweight web platform for online o^8^G prediction from user-submitted RNA sequences and (2) freely available source code and processed datasets. The web interface is hosted at www.rnamd.org/o8GPredictor, and the complete repository can be accessed at https://doi.org/10.6084/m9.figshare.29634239.v1.

## Results

### Baseline machine learning methods for o^8^G identification

To establish a prediction framework for o^8^G modification, we first evaluated several conventional machine learning approaches, including CNN (convolutional neural network),5^50^ SVM,5^51^ Random Forest (RF),5^52^ XGBoost (eXtreme Gradient Boosting),5^53^ and logistic regression.5^54^ Benchmark analysis (Table 2) demonstrates that all models achieved competent prediction accuracy (ranging from 0.88 to 0.93) and area under the curve (AUC) values (>0.93), confirming the learnability of o^8^G site prediction from sequence-derived features. Notably, XGBoost and logistic regression achieved the best overall performance in all five evaluation metrics, with XGBoost achieving the highest performance in accuracy (0.9258), AUC (0.9443), and F1-score (0.9188). Interestingly, while RF demonstrated superior precision, it performed less competitively in sensitivity and F1-score, indicating that the RF model tends to miss a significant portion of experimentally validated o^8^G sites. Although these results validate the feasibility of conventional approaches, they lack the capacity for deeper contextual understanding of nucleotide sequences. This motivated the development and fine-tuning of advanced pre-trained language models, which not only surpassed baseline performance but also enabled extraction of biologically interpretable sequence features.

**Table 1:** Overview and design of pre-trained foundation models

Model architecture	Pre-training corpus	Tokenization	Input k-mer size	Task head	Contextual info
BioBERT^55^	PubMed abstracts & PMC full-text	WordPiece	1-mer	2-class classifier	biomedical texts
BERT^56^	English Wikipedia & BooksCorpus	WordPiece	1-mer	2-class classifier	general language
DNABERT^43^	human genomic DNA sequences	k-mer (6-mer)	6-mer	2-class classifier	genomic sequence aware
RNABERT^57^	transcriptomic RNA sequences	k-mer (3-mer)	3-mer	2-class classifier	RNA-specific language

**Table 2:** Performance evaluation of different machine learning approaches for o^8^G identification

Model	Accuracy	AUC	Sensitivity	Precision	F1 score
CNN	0.8859	0.9377	0.7864	0.9746	0.9804
SVM	0.9092	0.9433	0.8250	0.9864	0.8985
Random Forest	0.8992	0.9338	0.7932	0.9794	0.8847
XGBoost	0.9258	0.9443	0.8614	0.9844	0.9188
Logistic regression	0.9203	0.9416	0.8568	0.9767	0.9128

### Overall performance of OBOE models

To optimize the OBOE model architecture, we developed four foundation-model-based framework and evaluated their performance on the prediction of o^8^G modification. The prediction performance was rigorously assessed using metrics including accuracy, area under the ROC curve (AUC), sensitivity (recall), precision, and F1-score. The detailed results are shown in Table 3. The architecture based on RNA-BERT demonstrated superior overall performance, outperforming the best baseline model XGBoost with significant improvement of 8.89% in sensitivity, 4.89% in accuracy, and 4.14% in AUC. The remarkable enhancement in sensitivity (0.8614–0.9455) is particularly important for comprehensive identification of validated o^8^G sites in biological applications. The DNABERT architecture also showed enhanced performance, balancing high sensitivity (0.9262) and precision (0.9787), to obtain an F1-score of 0.9517. Interestingly, the performance results indicated that the prediction architecture based on BERT2Ome exhibited relatively weaker performance across all evaluation metrics. These discrepancies among foundation-based models may be attributed to their distinct pretraining strategies and architectural specializations. Specifically, foundation models pre-trained on RNA sequences (RNABERT) or genomic k-mers (DNABERT) are substantially more effective at capturing the sequence patterns associated with o^8^G modification compared to general language models that lack biological sequence specificity or those pre-trained on biomedical text corpora, which cannot directly translate to RNA sequence pattern recognition. Consequently, the results led us to select RNABERT architecture for o^8^G sequence feature extraction.

**Table 3:** Performance evaluation of foundation-based framework

Model	Accuracy	AUC	Sensitivity	Precision	F1 score
Biobert	0.8947	0.8918	0.7836	1.0000	0.8787
Bert	0.9169	0.9172	0.8411	0.9922	0.9104
DNAbert_6mer	0.9535	0.9905	0.9262	0.9787	0.9517
RNAbert	0.9734	0.9851	0.9455	1.0000	0.9720

### Model interpretation of biological relevance generated by OBOE

**Figure 2:**
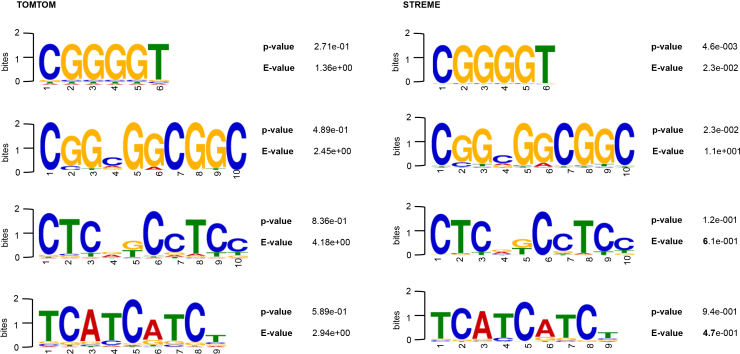
Motif analysis by TOMTOM comparison and STREME discovery Left column (TOMTOM): query motifs derived from the model or dataset were compared against a reference motif database using TOMTOM. Each sequence logo represents the most significant match reported by TOMTOM, with the height of the letters indicating the information content at each nucleotide position. The figure illustrates representative best matches for each query motif. Right column (STREME): high-contribution or positive sequences identified by the model were subjected to *de novo* motif enrichment analysis using STREME. Each sequence logo displays the nucleotide preferences and information content (bits) of the corresponding *de novo* motif, reflecting statistically enriched sequence features. The concordance between the two approaches (e.g., recurrent GC-rich or CTC-like patterns) indicates that the discriminative features captured by the model are biologically relevant. Specifically, STREME highlights statistically enriched *de novo* motifs, while TOMTOM maps these motifs to known factors or database entries, thereby facilitating biological interpretation.

To evaluate the consistency and biological relevance of the motifs identified by different approaches, we compared motifs derived from TF-MoDISco4^47^ (based on model contribution scores) with motifs discovered by STREME4^48^ (based on enriched sequence patterns) using the TOMTOM tool. To further investigate the sequence features underlying o^8^G peak predictions, we implemented an attribution-based interpretation pipeline using the Integrated Gradients (IG) method to assign base-resolution contribution scores for each input sequence (Figure 2). We employed TF-MoDISco,4^47^ which allows clustering of important k-mers and alignment based directly on contribution scores rather than hard-thresholded base selection. The computed attribution maps highlighted nucleotides with strong predictive importance, enabling the identification of context-dependent sequence determinants of o^8^G modification. High-scoring subsequences were then clustered and summarized using TF-MoDISco, which groups k-mers based on contribution patterns rather than raw sequence similarity, providing a more functionally relevant motif representation. The resulting motifs were visualized as sequence logos, revealing recurrent GC-rich patterns consistent with the chemical nature of o^8^G as an oxidative modification of guanine. Notably, while many guanine residues contributed positively, some exhibited negative or negligible contributions, suggesting that the predictive signal depends on sequence context rather than individual base identity alone.

The comparison between TF-MoDISCo and STREME-derived motifs revealed notable overlaps. Several TF-MoDISCo motifs exhibited strong matches to GC-rich elements identified by STREME (Figure 2), indicating convergence toward common sequence features associated with o^8^G modification. Such repetitive GC-rich motifs or CTC-like patterns are likely to promote stable RNA secondary structures, which may modulate the accessibility of guanine residues for oxidative modification. Additionally, CTC-rich elements have been implicated in RNA-protein interactions and alternative splicing regulation, suggesting that o^8^G deposition could be influenced by structural constraints and RNA-binding protein binding events. These observations highlight that our proposed OBOE captures not only local guanine enrichment but also higher-order sequence patterns, reinforcing the complementarity of deep-learning-based attribution methods and traditional motif discovery approaches in uncovering shared and biologically relevant determinants of o^8^G modification.

### Transcriptome distribution and functional characterization of o^8^G modification sites

**Figure 3:**
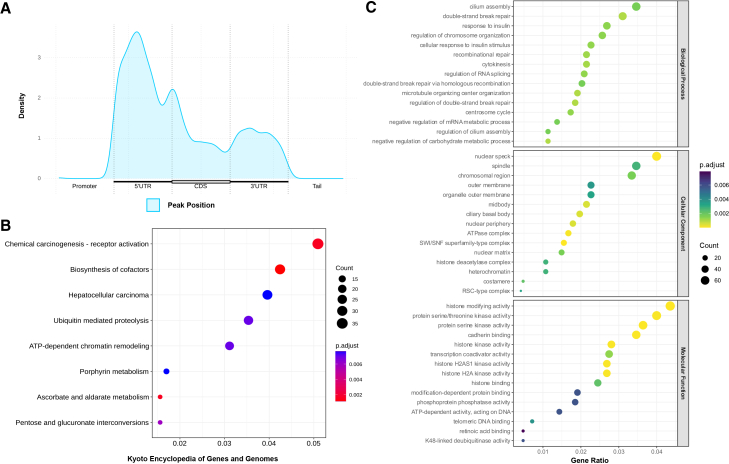
Functional characterization of o^8^G modification (A) Transcriptome-wide distribution of o^8^G modifications as revealed by MetaTX analysis. The positional preference of o^8^G across distinct transcript regions (5′ UTR, CDS, and 3′ UTR) highlights potential regulatory roles of this modification in post-transcriptional control. (B) KEGG pathway enrichment analysis of o^8^G-carrying genes demonstrates significant enrichment in pathways associated with cellular remodeling and metabolic regulation, suggesting that o^8^G may influence core cellular functions. (C) Gene Ontology (GO) enrichment analysis of o^8^G-carrying genes, categorized into biological process (BP), cellular component (CC), and molecular function (MF), provides insights into the broader biological contexts in which o^8^G exerts its impact. Collectively, these analyses support the functional relevance of o^8^G in shaping gene expression and cellular physiology.

To investigate the transcriptome distribution and functional implications of o^8^G RNA modification, we performed *Meta*-transcriptomic (MetaTX) analysis5^58^ on o^8^G-containing peaks across transcripts (Figure 3A). The results revealed a pronounced enrichment of o^8^G modification in the 5′ untranslated region (5′ UTR), suggesting a potential role in translation initiation and ribonucleoprotein complex assembly. Moderate enrichment was also observed in the coding sequence (CDS) and 3′ UTR, indicating possible involvement in translation elongation and mRNA stability. In contrast, o^8^G modifications were relatively scarce in the promoter region and transcript tail, implying a limited role in transcription initiation or polyadenylation processes.

To further elucidate the biological functions associated with o^8^G, we conducted functional enrichment analyses, including Gene Ontology (GO) enrichment and Kyoto Encyclopedia of Genes and Genomes (KEGG) analysis (Figures 3B and 3C**)**. KEGG pathway enrichment (Figure 3B**)** revealed that o^8^G-carrying genes were significantly enriched in pathways related to ubiquitin-mediated proteolysis, ATP-dependent chromatin remodeling, and hepatocellular carcinoma, as well as in several key metabolic processes, including ascorbate and aldarate metabolism and porphyrin metabolism. These pathways are well known for their roles in protein homeostasis, chromatin accessibility regulation, and metabolic reprogramming, processes frequently hijacked in cancer progression and cellular stress responses.^59^^,^^60^^,^^61^ GO enrichment analysis (Figure 3C) further supported the involvement of o^8^G in core regulatory processes. In the Biological Process (BP) category, o^8^G-carrying genes were significantly enriched in chromatin remodeling, double-strand break repair, and regulation of mRNA metabolic processes, suggesting a role for o^8^G in maintaining genomic integrity and in modulating RNA turnover, potentially via recruitment of specific RNA-binding proteins or reader complexes.1^1^^,^^2^ In the cellular component (CC) ontology, genes localized to nuclear structures such as the nuclear speck, SWI/SNF superfamily-type complex, and spindle apparatus, indicating a potential association of o^8^G with nuclear RNA processing centers and chromatin remodeling hubs.6^62^ In the molecular function (MF) domain, there was marked enrichment for histone-modifying activity, serine/threonine kinase activity, and RNA binding. These functions are essential in the regulation of transcription and RNA stability, further suggesting that o^8^G may contribute to transcriptome remodeling through cross-talk with histone marks and RNA-binding effector proteins.^63^^,^^64^^,^^65^ Collectively, these findings suggest that o^8^G is not randomly distributed but preferentially localized to functionally significant transcript regions, particularly the 5′ UTR, and may serve as a regulatory mark involved in translation control, chromatin dynamics, and stress-responsive signaling pathways.

### Web server implementation

**Figure 4:**
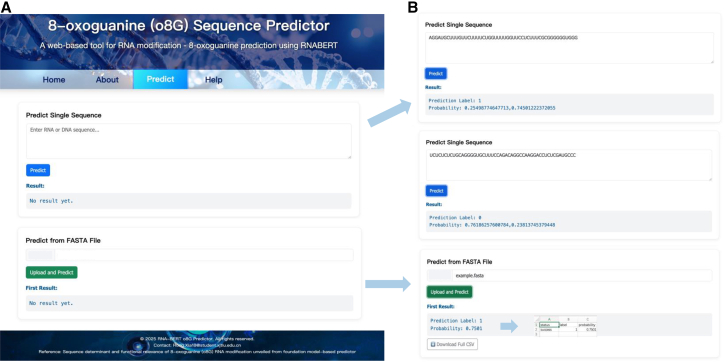
Web server interface and prediction output of the OBOE framework for o^8^G site identification (A) Screenshot of the web server input interface. The upper panel allows users to submit a single RNA sequence directly through the text input box, while the lower panel supports batch submission via FASTA file upload. After submission, sequences are processed by the underlying prediction model implemented in the OBOE framework. (B) Examples of the prediction output generated by the server. For each query sequence, the server returns a prediction label (1 indicating an o^8^G site and 0 indicating a non-o^8^ G site) together with a probability score representing prediction confidence. For batch submissions, the prediction results are summarized in tabular format and can be downloaded as CSV files for downstream analysis. This design enables convenient and scalable access to the OBOE prediction tool for transcriptome-level sequence screening.

To facilitate user accessibility, we implemented a dedicated web-based interface for OBOE, the o^8^G site prediction framework. The platform is organized into four main sections: Home, About, Predict, and Help, which offers step-by-step guidance for users. Within the Predict page, users can either input a single RNA sequence or upload a FASTA file for batch predictions. Upon submission, the server outputs the prediction label (0: non-o^8^G site; 1: o^8^G site) together with probability scores that reflect the prediction confidence. For batch submissions, results are provided in tabular format and can be directly downloaded as CSV files for downstream analyses. Detailed instructions for operation and additional technical information are available directly within the web interface. An illustrative example of the Predict page and its output interface is shown in Figure 4, demonstrating both the input options and representative prediction results.

## Discussion

Emerging evidence underscores the critical functional importance of o^8^G modification in regulating diverse cellular processes, including cancer development. The development of accurate computational approaches for the precise identification of o^8^G modifications is therefore crucial for facilitating their functional exploration. In this study, we made several key contributions. First, we introduce OBOE, the first computational tool for predicting RNA 8-oxoguanine sites, constructed by fine-tuning multiple pre-trained language models, including DNABERT, RNABERT, BERT, and BioBERT, thereby enabling effective embedding of RNA sequence information and identification of modification patterns. Using this framework, we discovered GC-rich and CTC-like sequence motifs that are potentially involved in oxidative stress regulation, providing mechanistic insights into *cis*-regulatory elements associated with o^8^G deposition. The enrichment of GC-rich sequence contexts is biologically plausible because guanine has the lowest redox potential among the four nucleobases and is therefore particularly susceptible to oxidative damage induced by ROS.^66^ GC-rich regions are also frequently associated with stable RNA secondary structures, which may influence local accessibility to oxidative modification. The observed CTC-like motif may reflect sequence environments associated with regulatory or structural constraints in RNA transcripts. Because oxidative stress and RNA dysregulation are linked to cancer-related processes,^67^ these motifs may provide preliminary clues regarding sequence contexts of o^8^G deposition, although further experimental validation is required. Additionally, we developed a user-friendly web platform to provide convenient access for sequence prediction and exploration, promoting broader adoption of our model in the research community.

Our systematic evaluation of 10 prediction frameworks, including five conventional machine learning methods and four pre-trained foundation models, demonstrated that foundation-model-based approaches consistently outperformed conventional methods. Notably, the RNABERT-based framework achieved the best performance, demonstrating its capability to effectively embed RNA sequence information and identify RNA modification patterns. To elucidate the sequence determinants of o^8^G modifications, we established an interpretability pipeline by integrating TF-MoDISco with established motif discovery tools (MEME and STREME). This analysis revealed several biologically relevant and evolutionarily conserved motifs in high-scoring regions, potentially representing *cis*-regulatory elements involved in o^8^G deposition. These findings provide a foundation for future mechanistic studies of oxidative RNA modifications.

However, three major limitations remain. First, the reliance on peak-based data may limit the generalizability of the models to genome-wide or transcriptome-wide contexts, particularly in less enriched or lower-signal regions. Second, while our models capture local sequence features, o^8^G modifications may also be influenced by other factors such as RNA secondary structure, oxidative stress status, or RNA-binding proteins, which were not incorporated into our current modeling. Third, experimental validation of predicted sites and motifs remains essential to confirm their functional relevance. Future studies could benefit from incorporating multimodal features such as RNA structure and protein-binding profiles. Furthermore, extending our framework to other oxidative or less-studied RNA modifications could provide new insights into the epitranscriptomic regulation. In conclusion, to our knowledge, our study represents the first application of fine-tuned pre-trained language models to o^8^G RNA modification, combining predictive modeling with mechanistic interpretation and making several key contributions, including the development of OBOE, identification of biologically relevant motifs, and creation of an accessible web-based platform, highlighting the feasibility of foundation models in epitranscriptomics and providing computational resources for the research community.

## Materials and methods

### Data collection and processing

In this study, we analyzed raw sequencing data generated by ChLoRox-Seq from BEAS-2B human lung epithelial cells under oxidative stress conditions. To identify transcriptome-wide o^8^G-modified regions, we employed the exomePeak2 pipeline with GC bias correction, which is better suited for detecting non-consensus, diffuse oxidative modifications such as o^8^G. Peak calling was performed using the default parameter settings of exomePeak2, and candidate peaks were retained using a false discovery rate (FDR) threshold of 0.05. Importantly, peak boundaries were determined dynamically based on read enrichment signals rather than a fixed window size, resulting in peaks with variable lengths across transcripts. In total, 2,356 high-confidence o8G peaks were extracted at exon-level resolution and used for subsequent model training and motif analysis.

To construct a balanced classification dataset, we employed a biologically informed strategy for negative sample generation. Specifically, we sampled guanine (G)-centered exon regions from annotated transcripts in the TxDb.Hsapiens.UCSC.hg38.knownGene database using the “*sample_sequence*” function from the m6ALogisticModel package to control for transcript-level sequence context and applied multiple filtering criteria: (1) location within exonic regions; (2) exclusion of any overlap with the identified o^8^G peaks; and (3) removal of sequences containing ambiguous bases (e.g., “N”). To ensure sequence context comparability, all regions were standardized to a fixed width corresponding to the median length of the detected o^8^G peaks, and 2,356 non-overlapping negative regions were randomly selected to maintain a 1:1 ratio with the positive set. For model development, both positive and negative samples were split into training (80%), validation (10%), and test (10%) sets while maintaining the 1:1 class ratio. Genomic sequences were extracted from the hg38 reference genome and converted into tokenized numerical representations for model input. To mitigate sequence redundancy and prevent model overfitting from highly similar sequences, we applied CD-HIT clustering6^68^ on the combined dataset. In addition to the primary dataset clustered at a sequence identity threshold of 0.9, we further constructed datasets with different sequence identity thresholds (100%, 90%, and 80%) to examine model robustness under varying levels of sequence redundancy. Using a sequence identity threshold of 0.9, we retained only representative sequences from each cluster, ensuring the training, validation, and test sets contained non-redundant examples. This filtering approach enhanced the sequence pattern diversity in the final dataset, and the consistent performance observed across datasets with different redundancy levels suggests that the predictive signals captured by the model are not solely driven by highly similar sequences, resulting in a more robust and generalizable model.

### Model architecture

To explore the sequence determinants of o^8^G RNA modification, we fine-tuned four BERT-style foundation models: BioBERT,5^55^ BERT,5^56^ DNABERT,4^43^ and RNABERT.5^57^ These models were selected for their distinct pre-training corpora and tokenization strategies, enabling a comprehensive evaluation of contextual and biological encoding. DNABERT was pretrained on genomic DNA sequences using k-mer tokenization and is well suited for capturing local nucleotide dependencies and regulatory sequence patterns. RNABERT was pretrained directly on RNA sequences and can encode RNA-specific contextual information, which is particularly relevant for RNA modification prediction. In contrast, BERT and BioBERT were included as general-language and biomedical-language baselines to evaluate the contribution of biologically informed sequence pretraining. Each model was adapted for binary classification by adding a fully connected prediction head. Input sequences were centered on the potential o^8^G site and tokenized using model-specific vocabularies (e.g., 6-mer for DNABERT, 3-mer for RNABERT). Training was conducted using the AdamW optimizer (learning rate: 2e−5), with a linear learning rate scheduler and warm-up steps. Models were trained using a cross-entropy loss function, and early stopping was employed based on validation performance. An overview of the model design and tokenization strategies is summarized in Table 1. While the architectures vary across models, all were fine-tuned using only raw RNA sequence inputs without any additional features. Among them, DNABERT achieved the best performance using 6-mer tokenization,4^43^ so we adopted the 6-mer input setting for comparison across all models. This standardized input scheme ensures a fair comparison by minimizing the influence of input granularity or feature engineering across models.

To illustrate the general fine-tuning strategy for large pre-trained sequence models used in this study, we highlight two representative examples: DNABERT and RNABERT. Both models are based on the Transformer architecture and adopt a tokenization-first approach, wherein biological sequences are segmented into overlapping k-mers prior to being embedded and encoded. Specifically, DNA-BERT (zhihan1996/DNA_bert_6) was pretrained on genomic DNA sequences with fixed 6-mer tokenization. We fine-tuned this model using a custom PyTorch loop, where k-mer-tokenized inputs were passed through a classification head and optimized with AdamW and a linear learning rate scheduler. In contrast, RNA-BERT (multimolecule/rnabert) was pretrained on large-scale RNA sequences using a masked language modeling (MLM) objective, capturing context-aware nucleotide representations suitable for RNA-related tasks. For fine-tuning, we trained the model using the Hugging Face Trainer framework with a binary classification head, optimized by AdamW. These two models exemplify the broader class of sequence-based pre-trained transformers leveraged in this study and were selected to compare how contextual representations learned from RNA or DNA corpora contribute to downstream prediction performance. The inclusion of diverse pre-trained encoders enables a systematic evaluation of model transferability across nucleic acid modalities. Detailed training hyperparameters for all evaluated models are summarized in Table S1.

### Model interpretation

To better understand the decision-making process of our predictive model, we conducted a systematic interpretability analysis aimed at identifying sequence features associated with RNA modifications. Specifically, we quantified the per-base contribution of input nucleotides using the IG method,6^69^ which computes the gradient of the output with respect to the input along a path from a baseline to the actual input. We implemented trapezoidal IG7^70^ to improve approximation accuracy and followed the suggestion by Kindermans et al.7^71^ to carefully select the reference input. Instead of using a zero or uniform background, we constructed a dinucleotide-shuffled version of the input sequence to serve as the reference, preserving local sequence dependencies and improving biological plausibility. The resulting saliency maps allowed us to visualize the importance of each base within the input sequence. To extract high-quality, non-redundant motifs from these importance scores, we adopted TF-MoDISco,4^47^ which identifies and clusters high-scoring subsequences across the dataset and generates consensus motifs that contribute most to the model’s predictions. While originally designed for TF binding analysis, TF-MoDISco is highly suitable for RNA modification tasks, with minor adjustments such as ignoring strand symmetry. These interpretation steps closely follow the strategy outlined in WeakRM for model interpretability and have proven effective in uncovering biologically meaningful sequence patterns learned by pre-trained models.4^40^ In parallel, we used STREME4^48^ to perform motif discovery directly from the labeled sequence data to identify statistically enriched motifs associated with modified sites. To validate the biological relevance of the motifs extracted through model interpretation, we conducted a comparative analysis using TOMTOM,4^49^ which aligns and matches the TF-MoDISco-derived motifs against the STREME-identified motifs. TOMTOM reports motif similarity statistics including *p* values, E-values, and Benjamini-Hochberg corrected q values to account for multiple hypothesis testing across motif comparisons. This comparison allowed us to assess whether the fine-tuned pretrained model had learned patterns consistent with statistically enriched sequence features, thus reinforcing the interpretability and biological validity of our approach. The full motif statistics generated by STREME and TOMTOM are provided in Tables S2 and S3.

## Data and code availability

The source code data are freely available at https://doi.org/10.6084/m9.figshare.29634239.v2 and the web interface is available at www.rnamd.org/o8GPredictor.

## Acknowledgments

This work was supported by the National Natural Science Foundation of China (32500580), the Natural Science Foundation of Jiangsu Province (BK20240723), the Scientific Research Foundation of Nanjing University of Chinese Medicine (013038030001), and the XJTLU Key Program Special Fund (KSF-E-51 and KSF-P-02). This work was also supported by the Supercomputing Platform of Xi’an Jiaotong-Liverpool University.

## Author contributions

R.X. conducted data processing, model construction, and analysis; developed the website; and drafted the manuscript. J.Z. performed the GO-related analyses. X.W. addressed issues related to the website. J. Ma and J.Y. provided guidance on biological aspects. P.W. and J.S. assisted with manuscript revision. D.H. contributed to resolving model-related questions. J. Meng conceived the overall framework of the study and revised the manuscript. B.S. provided the data and contributed to manuscript revision. B.S. provided the data and contributed to manuscript writing and revision.

## Declaration of interests

The authors declare no competing interests.

## Supplemental information

Document S1. Tables S1–S3

Document S2. Article plus supplemental information
