# Supplementary material for: Sequence determinant and functional relevance of 8-oxoguanine RNA modification unveiled from foundation-model-based predictor
Source: Mol Ther Nucleic Acids. 2026 May 14;37(2):102951. doi: 10.1016/j.omtn.2026.102951 (PMC13254886; doi:10.1016/j.omtn.2026.102951)
Supplement: Document S1. Tables S1–S3 [file mmc1.pdf]

OMTN, Volume *37*

Supplemental information

Sequence determinant and functional relevance of 8-oxoguanine RNA modification unveiled from foundation-model-based predictor

Rong Xia, Jiahao Zhang, Xuan Wang, Jiongming Ma, Jiayi Li, Jionglong Su, Prudence Wong, Daiyun Huang, Jia Meng, and Bowen Song

Supplemental Tables

Table S1. Training hyperparameters used for fine-tuning different language models

Parameter	RNABERT	DNABERT	BioBERT	BERT
**Backbone model**	multimolecule/rnabert	zhihan1996/DNA_bert_6	dmis-lab/biobert-base-cased-v1.1	bert-base-uncased
**Maximum sequence length**	256	512	512	512
**Batch size**	16	16	16	16
**Training epochs**	5	3	3	3
**Optimizer**	Hugging Face Trainer	AdamW	AdamW	AdamW
**Learning rate**	2e-5	2e-5	2e-5	2e-5
**Learning rate scheduler**	HF Trainer default	Linear scheduler	StepLR	StepLR
**Scheduler parameters**	epoch-based evaluation	warmup steps = 0	step_size = 3, gamma = 0.1	step_size = 3, gamma = 0.1
**Padding strategy**	max_length	max_length	max_length	max_length
**Sequence truncation**	Yes	Yes	Yes	Yes
**Data shuffling**	Yes	Yes	Yes	Yes
**Number of output labels**	2	2	2	2
**Model checkpoint strategy**	Best model at end	Final epoch model	Final epoch model	Final epoch model
**Random seed**	42*	42*	42*	42*

Note: The random seed was fixed during training to improve reproducibility.

Table S2. STREME-discovered motifs

Sequence motifs discovered from o8G-associated sequences using STREME. The table reports motif consensus sequences together with enrichment significance statistics (p-values and E-values) provided by STREME.

Motif ID	Motif shown in Fig.2	STREME consensus	STREME p-value	STREME E-value	Sites
**Motif 1**	CGGGGT	ACCCCG	4.6e-03	2.3e-02	68 (2.9%)
**Motif 2**	CGGCGGCGC	GCCGCCGCCG	2.3e-02	1.1e-01	78 (3.3%)
**Motif 3**	CTCGCCTCC	CTCRKCCTCC	1.2e-01	6.1e-01	147 (6.2%)
**Motif 4**	TCATCATC	TCATCATCT	9.4e-01	4.7e+00	64 (2.7%)

Table S3. TOMTOM motif similarity results

Motif similarity comparison results obtained using TOMTOM. For each discovered motif, the best matching motif in the reference database is shown together with the corresponding p-values, E-values, and Benjamini–Hochberg corrected q-values.

Motif ID	Best TOMTOM Match	p-value	E-value	q-value	Orientat-ion	Overlap
**Motif 1**	ACCCCG	2.71e-01	1.36e+00	1.00e+00	Reverse	6
**Motif 2**	GCCGCCKCCG	4.89e-01	2.45e+00	1.00e+00	Reverse	10
**Motif 3**	CTCRKCCTCC	2.07e-01	1.04e+00	1.00e+00	Normal	9
**Motif 4**	TCATCATCY	5.89e-01	2.94e+00	1.00e+00	Normal	8
